# Foot Pad Health as Part of On-Farm-Monitoring in Turkey Flocks

**DOI:** 10.3389/fvets.2019.00025

**Published:** 2019-02-19

**Authors:** Kathrin Toppel, Birgit Spindler, Falko Kaufmann, Matthias Gauly, Nicole Kemper, Robby Andersson

**Affiliations:** ^1^Faculty of Agriculture, University of Applied Sciences Osnabrueck, Osnabrueck, Germany; ^2^Institute for Animal Hygiene, Animal Welfare and Farm Animal Behavior, University of Veterinary Medicine Hanover, Foundation, Hannover, Germany; ^3^Faculty of Science and Technology, Free University Bozen-Bolzano, Bolzano, Italy

**Keywords:** foot pad dermatitis, turkey welfare, sample size, on-farm assessment, indicator, scoring system

## Abstract

Currently, there is no consistent approach to on-farm and post-mortem foot pad (FP) assessment in turkey husbandry in sampling of both feet, sample sizes of birds and scoring schemes during the production period. Therefore, in a field study, 11,400 turkeys, i.e., 22,800 feet, were macroscopically scored at 4-week intervals, 60 birds per flock per date, in accordance with the scale system of Hocking et al. (1). Spearman's rho was calculated between the foot pad dermatitis (FPD) score of both feet of an individual turkey. Sample size for FPD monitoring was calculated for several flock sizes, considering expected FPD prevalence and the error and confidence level (α = 0.01, 0.05, 0.1). To compare macroscopic to histological findings, ten excised FPs were histopathologically investigated by hematoxylin & eosin staining. To align manual macroscopic FPD evaluations with a technical system, 20 photographic images of FPD were measured using the ImageJ program. The scores of both feet of an individual turkey correlated between *r* = 0.252 and *r* = 1.000. Thus, both feet of a bird should be monitored, while the worse foot should be evaluated. As an exemplary sample size for on-farm FPD assessment, 77 turkey poults were calculated in a flock of 4,000 birds with an expected FPD prevalence of 40% and α = 0.1. The sample size of monitored birds within a flock should differ and depend on flock size and expected FPD prevalence. Histopathological findings showed normal and non-affected structures of a macroscopic Score 0 and a moderate ulcer of the macroscopic Score 1 and Score 2. The applied assessment scheme should distinguish first alterations and scar tissue as separate scores to differentiate the need for management intervention vs. the success of management measures that were already implemented. FPD affected areas were given lower Scores and assessed to be healthier when evaluated by an image system, compared to a manual assessment. Furthermore, with regard to an increase in camera-based assessments, the boundary of the metatarsal pad needs to be clarified. In conclusion, a new scoring system is required, as the size of the FP cannot be clearly defined and different tissue textures, as well as valid sample sizes are not currently sufficiently considered.

## Introduction

Foot pad dermatitis (FPD) is a major concern for poultry health and welfare in the European Union (2). It has already been proven that management has an influence on foot pad health (3–5). Therefore, foot pad health could be used as a suitable animal-based indicator for husbandry and environmental conditions. Foot pad health assessment has been specified by the European authorities as an option for broiler welfare assessment and it can be used in turkeys as well (6).

Several studies have proven a high prevalence of FPD in chickens (7) and in turkey flocks (8–10), including within the rearing period of turkey production (5, 11–13).

A redness and dark discoloration of scales are often a first indication of foot pad lesions. Additionally, rhagades herald the first signs of degenerative processes on the plantar surfaces of foot pads (14). These macroscopic findings are followed by hyperkeratosis and necrosis of the epidermis (15). Foot pads were examined microscopically, and inflammatory infiltration was detected on foot pads with macroscopically mild lesions (8, 11, 16). According to Spindler (8), it can be assumed that increased macroscopic alterations indicate a deeper lesion. The age of birds must be considered, as in younger turkeys the size of the lesions increases rather than lesion depth, as seen in older birds (17, 18). FPD can lead to the irritation of sensitive nerve endings in the dermal tissues causing pain, harm, and discomfort (11).

Foot pad lesions can completely heal (3, 17). According to Platt (18), scars are visible due to an eliminated scale structure and a pale and even foot pad. The author also stated that week 14 to 21 of life, represents a good healing potential window (18). A period of 15 days for healing and scar formation was observed by Mayne et al. (3).

As an example, to use foot pad health as an animal-based indicator for husbandry and environmental conditions, within German turkey production, a benchmark system has been established between batches within a slaughterhouse. The benchmark is based on several animal-based indicators, e.g., foot pad health. Within that system, foot pad assessment follows the European 5-point foot pad scoring system from Hocking et al. (1). The three categories, A, B and C, conform to 0 or 1 (A), 2 and 3 (B), and 4 (C). The latter category should identify conspicuous flocks (19). Several foot pad scoring systems for the turkey species, based on macroscopic (1, 3, 20–22) and histological (11) findings have been investigated and published in the past. Bergmann et al. (13) evaluated foot pad health during the rearing period by modifying the scoring systems of Hocking et al. (1) and Mayne et al. (3). The assessment schemes show differences, e.g., the number of scales. Furthermore, proposed sample sizes in field studies differed between 50 (23, 24) and 60 randomly selected birds per flock (5, 13). A difference in expected prevalence was not considered within the recommended sample sizes (24), whereas this was calculated for a post-mortem evaluation scheme (1).

Alongside manual assessment at the slaughterhouse, a camera system was also established for post-mortem evaluation. The system calculates the percentage of an affected black (i.e., necrotic) area on the foot pad [CLK Turkey Check; (25)] or the size of a necrotic area, independently from the size of the foot pad [MEYN Foot pad Inspection System; (26)]. The latter assessment system would offset the lack of an anatomic definition of the foot pad area for macroscopic assessment. Lund et al. (27) investigated the manual evaluation compared to a camera-based evaluation of broiler foot pads at the slaughterhouse. The assessment was based on a 3-point scale and raters tended to choose the middle score as the most frequent category, whereby results from camera-based evaluation recorded more data in the worst category. Lund et al. (27) derived that foot pad dermatitis seems to be underestimated.

The increased relevance of FPD scores as measured by future compulsory manual assessments and particularly via automated camera systems requires a consistent approach for on-farm and post-mortem foot pad assessment.

This paper aims to address the matters outlined above in three different sections:

Section 1: Foot pad data from a field study were investigated to check and separate the prevalence of no lesions and mild lesions, occurring during the rearing period. The dispersion of the affected feet of a single bird were calculated. The necessary sample size for macroscopic foot pad assessment was calculated, considering the expected prevalence, and flock size as well as different levels of confidence, to enable flock-specific actions during the rearing and fattening periods.

Section 2: Macroscopically scored foot pads were compared with histological findings.

Section 3: The necrotic area of foot pads was macroscopically calculated by an imaging program to indicate possible differences between technical and manual observations regarding the extent of alterations on the metatarsal pad.

## Section 1—Macroscopic Investigations Concerning the Following Issues: FPD Prevalence, Macroscopic Evaluation, Number of Affected Feet of a Single Bird and Sample Size of FPD Assessment

### Materials and Methods

#### Birds and Husbandry

A field study was conducted on 13 commercial turkey farms, over a 1-year period. Foot pad data from two consecutive production cycles per farm were collected (170,000 toms and 37,000 hens in total). On-farm data were monitored at 4-week intervals in 30 male and seven female flocks on four rearing farms, four fattening farms and five combined farms. On rearing farms, day-old turkey poults were kept until an age of 35 days (rearing period) and then rehoused for the fattening period (day 35 until slaughter). On combined farms, birds remained in the rearing barn during the fattening period. A flock was defined as a group of animals placed in the same barn. The major genetic brand was B.U.T. 6 (31 flocks), followed by B.U.T. 7 (four flocks), and B.U.T. TP7 (two flocks). The average rearing period was 31.2 (±3.4) days. The fattening period for hens was 113.6 (±2.1) and 145.8 (±3.1) days for toms, respectively.

#### Procedures and Observations

The foot pads of 60 randomly selected birds per flock and barn were scored in the 1st, 4th, 8th, 12th, and 16th week, as well as post-mortem in male flocks (only). The plantar area of both foot pads of individual birds were macroscopically scored. During the rearing phase, the foot pad scale of Bergmann et al. (13) was adopted. The five categories for assessing reared birds were Score 0 = no alterations on the surface of foot pad; Score 1 = hyperkeratosis, moderate hypertrophy of the plantar skin, no dark colored but elongated reticulate scales, Score 2 = severe hyperkeratosis, pronounced hypertrophy of the plantar skin, adhesive dirt cannot be removed without damaging the skin of plantar surface; Score 3 = superficial lesions, epithelial necrosis, dark-colored necrosis of (elongated) reticulate scales; Score 4 = foot abscess, ablation of the outer layer of the epidermis.

During the fattening period, the 5-point scale of Hocking et al. (1) was used: Score 0 = no external alterations; Score 1 = harder and denser foot pad with raised center, small necrotic areas, and separated reticulate scales; Score 2 = marked swelling of foot pad, necrotic area covering less than a quarter of foot pad; Score 3 = evident swelling, enlarged foot pad size, pronounced, and separated reticulate scales, necrotic area covering up to half of foot pad; Score 4 = as Score 3, necrotic area covering more than half of the foot pad.

The depth of a lesion was not recorded. To evaluate the prevalence and severity of FPD, both feet of an individual were monitored and the worst foot (highest score) of a bird was evaluated.

### Statistical Analysis

The inter-observer consistency in farm scoring, between the two observers who monitored foot pad health in this project, was checked before data collection. To do so, 200 pairs of foot pads from 200 turkeys were scored and Kendall's- tau- b was calculated (*r* = 0.949; *p* < 0.01).

The Spearman's correlation coefficient was calculated by SPSS Vs.24 with a confidence level of 0.95 for the right and left foot of a pair separated by sex, and also for individual flocks.

The sample size was calculated using a standard statistical method for epidemiological studies [(28); see also (1)]. The expected prevalence (of foot pad lesions/dermatitis), expected error, and confidence level were considered.

$$\begin{array}{l} The\;equation\;is\;n=\frac{N*Z^{2}*P\left(1-P\right)}{d^{2}*\left(N-1\right)+Z^{2}*P\left(1-P\right)} \end{array}$$

The proposed standard was calculated using the formula n = Z^2^^*^P^*^(1–P)/d^2^, with n = sample size, N = flock size (number of birds), Z = z-value, P = Prevalence (% affected birds of a flock), d = α-error or confidence level.

The sample sizes for prevalence figures of 10, 20, 30, 40, and 50% were calculated, also considering an error rate of 0.01, 0.05, and 0.1 with 99, 95, and 90% confidence for flocks of different sizes with 1,000–10,000 birds.

### Results

The results of the evaluation of foot pad health during the rearing and fattening periods, showed major differences between the alterations in the right and left foot of an individual over time. The example of a female flock in Table 1 shows a correlation between both feet of a pair of *r* = 1.000 within the first week and *r* = 0.401 within the fourth week of life and finally *r* = 0.252 at the end of the fattening period. Therefore, high variances within flocks were possible.

**Table 1:** Correlation between the right and left foot of an individual bird (r = bold values); flocks divided by sex (summarized sample 11,400 pairs from 13 farms = 37 flocks).

**Determinants**	**Week of Life**					
	**1**	**4**	**8**	**12**	**16**	**p.m**.
Male (wc)	**0.691**	**0.730**	**0.355**	**0.644**	**0.664**	**0.566**
*n*	450	600	600	810	840	840
Male (sc)	**0.677**	**0.667**	**0.522**	**0.473**	**0.621**	**0.502**
*n*	600	660	960	960	960	1,170
Female (wc)	**0.741**	**0.550**	**0.364**	**0.686**	**0.548**	No evaluation
*n*	210	240	240	240	240	
Female (sc)	**1.000**	**0.357**	**0.398**	**0.520**	**0.526**	No evaluation
*n*	120	180	180	180	120	
Sample- single male flock	**0.615**	**0.790**	**0.693**	**0.679**	**0.383**	**0.531**
*n*	60	60	120	180	180	180
Sample- single female flock	**1.000**	**0.401**	**0.323**	**0.562**	**0.252**	No evaluation
*n*	60	60	60	60	60	

*(Spearman correlation, wc, winter cycle; sc, summer cycle)*.

**Figure 1:**
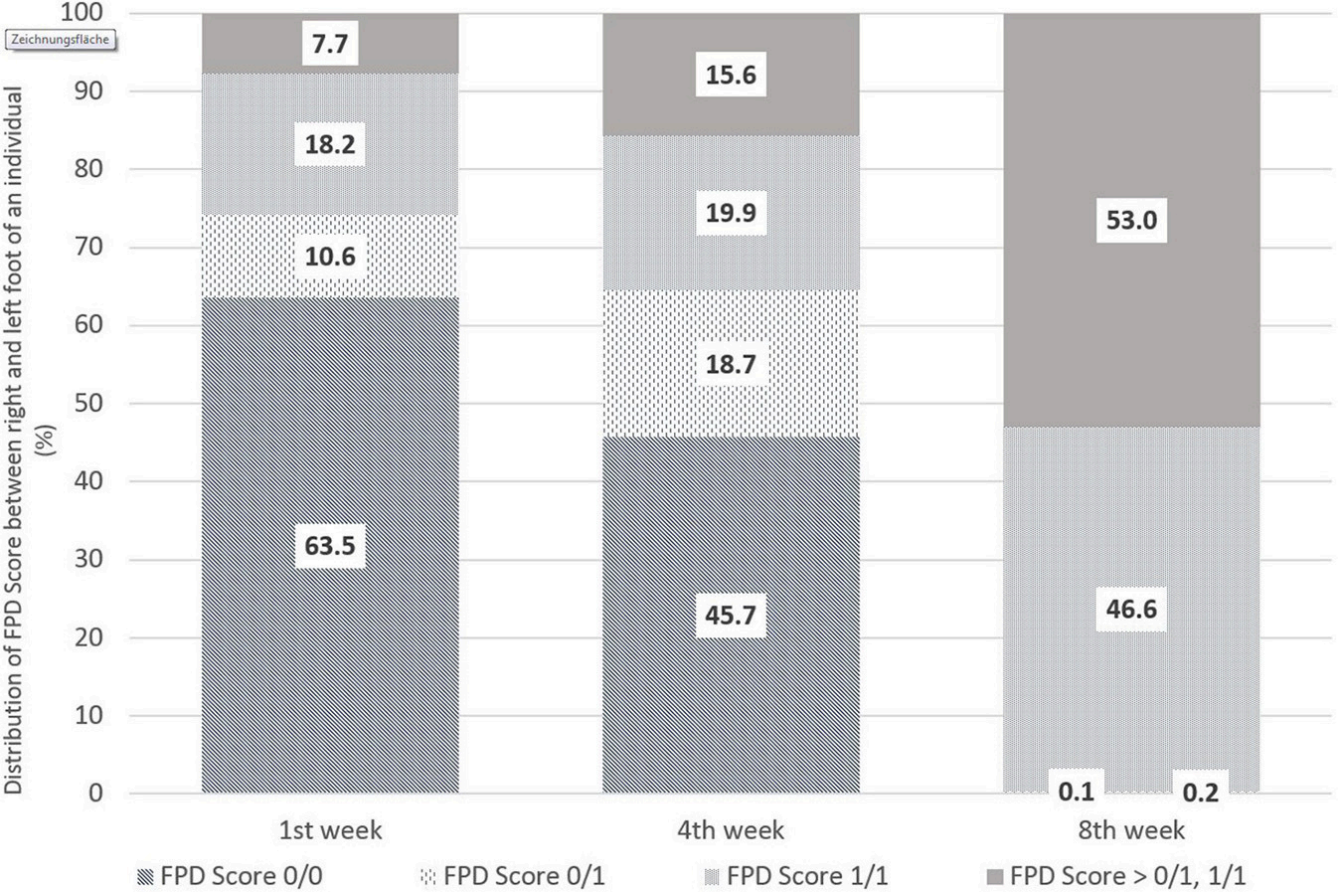
Comparison of FPD-Scores between the right and left foot of an individual bird from the first to eighth week of life in % [*n* = 1,382 pairs (1st week), 1,681 pairs (4th week), 2,013 pairs (8th week)] (where Score 0/0 means no FPD on left and right foot; 0/1 one foot of the pair with FPD Score 1 and the other Score 0, 1/1 both feet with FPD Score 1 and >0/1;1/1 stands for at least one foot of an individual worse than Score 1).

Figure 1 shows the distribution of foot pad scoring between the left and right foot of an individual, in particular with regard to Score 0 and Score 1. The evaluations were performed in the 1st, 4th, and 8th weeks of life. Within the first week, 63.5% of the pairs were scored without any lesions on the left and right foot (0/0). Inversely, in 35% of the pairs of foot pads evaluated, at least one foot differed from Score 0 (0/1, i.e., one of the two feet of an individual showed FPD Score 1 and one no FPD; 1/1 both feet with FPD Score 1; > 0/1, 1/1 means at least one foot of an individual worse than Score 1). The proportion of pairs without any alterations decreased to 45.7% by week 4 and to 0.1% in week 8. Pairs of feet with a FPD Score > 0/1, 1/1 were proportionally the highest in week 8 with 53.0%.

To estimate foot pad health within a flock, the sample size for monitoring was calculated. Suggested sample sizes increased with the level of confidence, as shown in Table 2. A symmetrical calculation with an identical sample size leads to a prevalence of 90, 80, 70, and 60%, which is equal to 10, 20, 30, and 40% (non-) affected birds, respectively [cf. (1)]. As an example, the monitoring of the status quo of FPD in a flock of 4,000 birds per barn unit, requires a sample size of 77 birds if the expected FPD prevalence is about 40% (or vice versa 60% non-affected birds) during rearing with a confidence level of 95%. The expected value would be adjusted to the data of evaluation in Table 1. Furthermore, a higher expected FPD prevalence during fattening would require a smaller sample size for FPD monitoring. An expected prevalence of 90% would require assessment of a total of 29 birds. A higher confidence interval of 99% would be realized by a higher sample number.

**Table 2:** Calculated number of birds to be monitored and evaluated depending on flock size, prevalence of footpad alterations, confidence interval (α 0.1 = 90%, 0.05 = 95%, and 0.01 = 99%, respectively), and proposed standard.

**α of FPD expected frequency**	**FPD expected frequency (%)**	**Flock size**										**Proposed Standard**
		**1,000**	**2,000**	**3,000**	**4,000**	**5,000**	**6,000**	**7,000**	**8,000**	**9,000**	**10,000**	
0.1	10	29	29	29	29	29	29	29	29	29	29	30
0.1	20	50	51	52	52	52	52	52	52	52	52	52
0.1	30	65	67	67	68	68	68	68	68	68	68	69
0.1	40	68	73	76	77	77	78	78	78	78	78	79
0.1	50	76	79	80	80	81	81	81	81	81	81	82
0.05	10	124	132	135	136	137	138	138	139	139	139	141
0.05	20	201	223	232	236	239	241	242	243	244	245	251
0.05	30	248	283	297	304	309	312	315	316	318	319	329
0.05	40	274	317	334	344	350	354	357	359	361	363	376
0.05	50	282	328	347	357	364	368	371	374	376	377	392
0.01	10	823	1,398	1,823	2,149	2,408	2,618	2,792	2,939	3,064	3,171	4,644
0.01	20	892	1,610	2,201	2,695	3,114	3,475	3,788	4,063	4,306	4,523	8,256
0.01	30	916	1,689	2,350	2,922	3,422	3,862	4,253	4,602	4,917	5,201	10,836
0.01	40	925	1,722	2,415	3,024	3,562	4,042	4,472	4,861	5,212	5,533	12,384
0.01	50	928	1,732	2,434	3,053	3,604	4,095	4,538	4,938	5,302	5,633	12,900

### Discussion

The results during rearing show a correlation coefficient between the right and left foot of an individual equal to or <*r* = 0.790. Other authors achieved results of ~*r* = 0.830 (Spearman's rank correlation coefficient; 11,830 pairs) (9) and *r* = 0.835 (Spearman) (13). Based on the study of Krautwald-Junghanns et al. (9), Allain et al. (10) evaluated only the right foot of an individual in their study. This method was also applied in the study by Bergmann et al. (13). However, in considering of the welfare of an individual, and by using a small sample size, more precision can be achieved by monitoring both feet of a single turkey and evaluating the ones classified as being most severe during an on-farm evaluation. This conclusion was also reached by Hocking et al. (6), as they only evaluated the worst foot pads in their study, which is also recommended by Knierim et al. (24).

The first alterations on metatarsal pads were observed within 7 days post hatching. These findings are comparable to those of Bergmann et al. (13). The present results show the necessity for early monitoring and evaluation during the rearing period, as well as the use of a scoring system allowing the evaluation of first alterations separate from non-affected feet or no lesions (Score 0). This is in contrast to recommendations for a self-monitoring system by Knierim et al. (24). The latter authors suggest an assessment during the fifth week of age, based on a scheme which includes no lesions and small necrotic areas within the same scoring category. In this context, evaluating the foot pad with the highest score [worst performance; c.f. (13, 24)] would reflect the realistic situation of the flock.

The calculation of the sample size was conducted in accordance with Hocking et al. (1). The authors calculated a prevalence of FPD beginning with necrotic areas in accordance with Score 2 (necrotic area up to a quarter of the foot pad) on the 5-point scale at slaughter. In contrast to that scheme, on-farm monitoring should consider all lesion formations in order to signal the beginning of FPD and also the development of foot pad alterations. The sample size used might be a compromise between estimating FPD prevalence and the economic feasibility of monitoring, as well as minimizing stress for the birds caused by handling and lifting the individuals. To meet the latter needs and to benefit from on-farm monitoring, it is probably most realistic to assess on the basis of a 90% confidence level. Additionally, references of FPD prevalence are available from former flocks (29). Moreover, camera-based assessment of foot pad health is increasing and allows a much higher sample size post-mortem. De Jong (30) described 96.2% of feet being assessed daily at a broiler slaughterhouse via the Meyn Foot Pad Inspection System. Depending on the technical precision and accuracy of the assessment method at slaughter, as well as the large sample size, a monitoring system based on one foot of an individual is deemed to be most suitable.

## Section 2—Histological Investigations to Assess the Depth of Macroscopic Lesions

### Materials and Methods

A total of 30 feet from male turkeys at the end of fattening (21st week of life) were first scored macroscopically and then a histopathological investigation of ten excised foot pads (macroscopically scores 0–4 and a bumble foot) was performed. This method required tissue from the center of the metatarsal pad to be removed and fixed in formic acid (10%) for 24 h for histological examination. Afterwards, slices were constructed with a standard microtome of about 5 μm and were stained with hematoxylin/eosin (HE). After processing, the histological samples were examined under a light microscope, evaluating the histopathological characteristics of the epidermis (*Stratum corneum* and *Stratum profundum)* and dermis (*Corium*) based on the arrangement of the scales [carried out by LAVES, Oldenburg, in accordance with Mayne et al. (3) and Spindler (8)]. Characteristics of lesions were separated according to occurrence and severity of slight, moderate or severe hyperkeratosis, erosion and ulceration. A further parameter was indicative of an inflammatory process, proven by an infiltration of granulocytes and the presence of bacteria.

### Results

A sample of six macroscopically different foot pads with histopathological findings is presented in Table 3.

**Table 3:** Examples of macroscopic and histological observations of foot pads with different levels of foot pad lesions.

**Macroscopical observations**	**Histopathological findings**
**SCORE 0**	
No external alterations on the surface of foot pad	Normal skin structure, epidermis with *stratum corneum* (St.c.) and stratum intermedium (St.i.) of normal thickness and dermis (d)
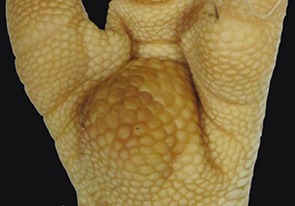	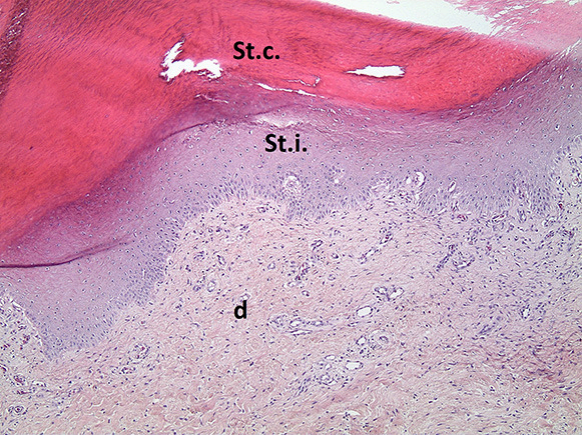
**SCORE 1**	
Harder and denser foot pad with raised center, small necrotic areas, and scar tissue with separated reticulate scales, no swelling	Moderate ulcer: necrosis (n) of epidermal and dermal structure; dermis (d) with scar tissue; moderate infiltration of granulocyte population in epidermis and dermis and cell detritus (cd)
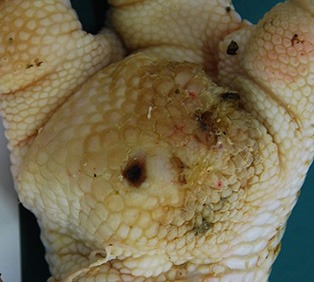	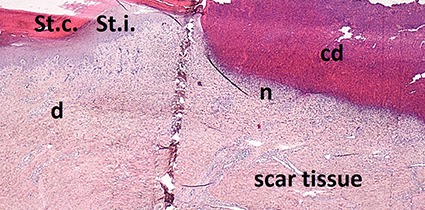
**SCORE 2**	
Marked swelling of foot pad, necrotic area <¼ of foot pad	Moderate ulcer: Necrosis (n) in epidermal and dermal (d) structure, moderate cell detritus (cd)
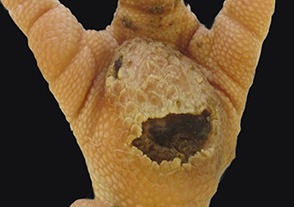	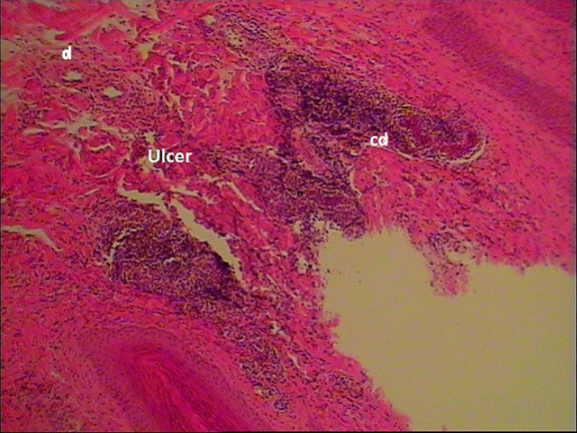
**SCORE 3 AND SCORE 4**	
Evident swelling, enlarged foot pad size, pronounced, and separated reticulate scales, necrotic area up to ½ (Score 3) or more than ½ (Score 4) of foot pad, respectively.Visible necrotic lesion, loss of epidermis, dark adherent crust, reticulate scales form a white boundary around necrotic area	Severe deep ulcer: Massive necrosis (n) in epidermal and dermal structure, cell detritus (cd), in dermis (d) massive migrated granulocyte, large-scale alterations
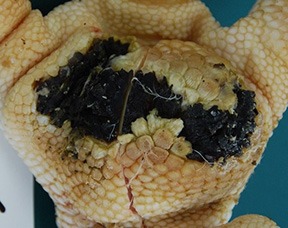 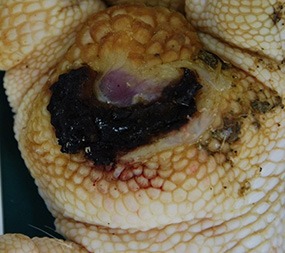	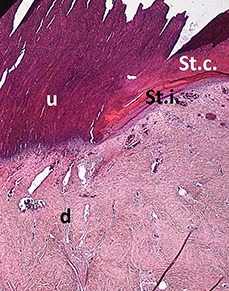 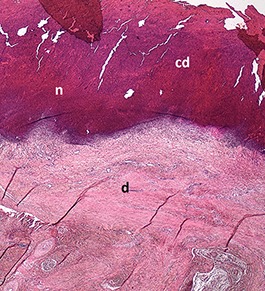
**BUMBLE FOOT**	
Swollen enlarged foot pad, necrotic area <½ of the foot pad due to swelling	Abscess, swollen collagen structure, clearly visible massive bacterial colonies in dermal structure
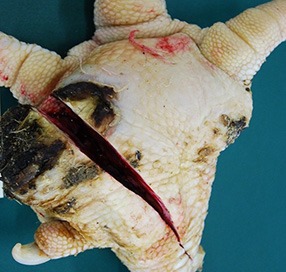	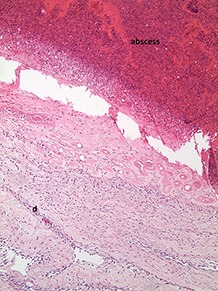

At the macroscopic foot pad Score 0 with no visual findings, a slight hyperkeratosis, characterized by an extension of the *Stratum corneum*, and the extension of the scales were discernible. At the macroscopic foot pad lesion Score 1 with no swelling, a slightly rough scale structure, scar tissue and a small necrotic area of metatarsal pad, were already present. The following Scores 2–4 resulted in a larger extension of the histopathological findings; for example, the size of the ulcerated area due to an inflammatory process. This macroscopic scoring indicated a large necrosis on the plantar area. This was covered by a dark adherent crust and showed a white boundary of reticulate scales around the necrosis.

The most affected foot displayed a swollen and enlarged foot pad, so-called bumble foot, whereby a necrosis was formed with a surface rigid to the touch. Histological findings showed a prominent abscess and a swollen collagen structure. Strong bacterial colonies were also detected.

### Discussion

The macroscopic scoring of the foot pad Score 1 showed a covering of a slightly rough scale structure. The development of the scale-shaped structure requires an intact dermal structure (18). The existence of a macroscopically small, affected, dark-colored area (Score 1) was proven histopathologically to herald the presence of an ulcer, where the epidermis and dermis were affected, and inflammatory tissue was present. Typically, cell detritus and necrosis developed under the plantar surface. Additionally, a granulocyte population was detected in the epidermis and dermis indicating inflammatory processes, in agreement with Spindler (8). This is also in accordance with studies performed on broilers, for example, Greene et al. (31), where the development of a severe ulcer in broiler foot pads within <1 week on a previously intact plantar surface was described. Heitmann et al. (32) also found ulcerations in a broiler foot pad which received a low score macroscopically. When taking up the assessments of Hocking et al. (6) and Spindler (8), it can be assumed that increased macroscopic alterations indicate a deeper lesion. Therefore, detecting first lesions separate from Score 0, is essential for implementing timely measures, in particular considering animal welfare.

In the case of ulcers and deep lesions, the affected dermis and epidermis can also recover. A white area is clearly visible on the surface and indicates scar tissue, which develops instead of reticulate scales (18, 33). Platt (18) described evidence of scar tissue at the end of the fattening period in most of the bird's foot pads in her study. A swollen and enlarged foot pad, so-called bumble foot, is formed by a prominent abscess and swollen collagen structure. The histological results correlated with the macroscopic findings. Bumble foot was described as causing pain, limited mobility, and reduced water consumption (34).

However, injured foot pads do not necessarily lead to deficiencies in gait and activity which therefore may not be used to indicate the presence of a foot pad problem (9). This emphasizes the necessity for the on-farm monitoring of foot pad health by picking out single birds and looking at the feet.

## Section 3—Comparison Between Camera-Based Macroscopic Assessment and Manual Evaluation

### Materials and Method

**Figure 2:**
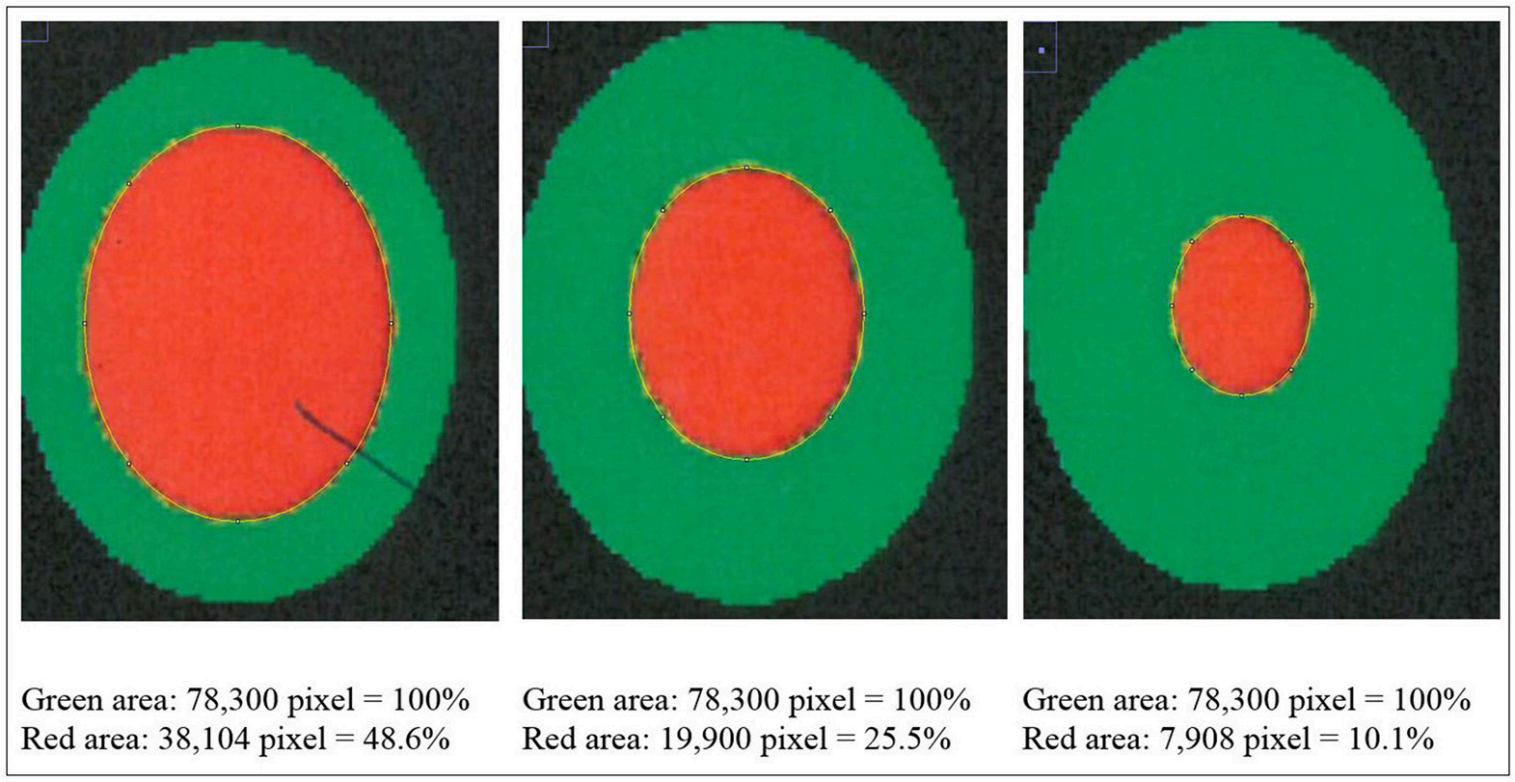
Surface ratio green = foot pad, red = necrosis, fine yellow frame presents manually marked area; output number of pixels by ImageJ 1.51 k.

The camera system used in German turkey slaughterhouses, which calculates the proportion of a necrotic area in relation to the estimated area of the metatarsal pad [CLK—Cruse Lappelmann Kognitionstechnik, Altenberge, cf. (25)], was evaluated in this study. To examine the optical illusion of altered (= necrotic) areas on metatarsal pads, human subjective evaluation was compared to a technical solution. Twenty randomly selected pictures from turkey foot pads and digits were analyzed. Foot pads had either identifiable, macroscopic necrosis on the plantar surface of the foot pad or digit or showed visible scar tissue. For estimating the surface ratio, the image processing system ImageJ 1.51 k (vs. 64bit, Bethesda, USA) was used. A certain area was marked and represented in a number of pixels (Figure 2). Visible alterations on turkey foot pad photographic images were tagged freehand, analyzed, and measured by the program. The dimensions of alterations were categorized according to the scheme of Hocking et al. (1) (see Materials and Methods in Section 1). Finally, information for herd managers was derived, depending on the evaluation at the slaughterhouse or the on-farm monitoring.

### Statistical Analysis

Each photographic image was measured with three replications. The coefficient of variation (CV) was calculated for every boundary (green, red and blue area) by SPSS Vs.24. The equation was CV = s/x, while s = standard deviation and x is sample mean.

### Results

Figure 2 shows representations of different altered areas which were presented. It might be assumed that a human observer would tend to give a higher or worse assessment, especially considering the picture on the left, compared to the technical value of 48.6% red area. In accordance with Hocking et al. (1), the photographic image on the left would be categorized as being equivalent to Score 3.

Several foot pad alterations were quantified in proportion to the foot pads area. The results are presented in Table 4. The first picture was obtained from the official European foot pad scoring system for turkeys in meat processing plants (1). The calculated area of a foot pad was given a green boundary, the red border marked the necrotic or black area. In accordance with Hocking et al. (1), the photographic image corresponds to Score 4, which is described with a necrotic area covering over 50% of the foot pad. Considering the presented assumption of the foot pad area, Figure 1 resulted in a 25.1% necrotic area, which is equivalent to Scores 2 or 3 on a camera-based evaluation at the slaughterhouse.

**Table 4:** Calculation and description of macroscopic foot pad alterations [green line: 100% metatarsal pad; red line: detected necrotic (black) area: blue line: scarred tissue; three samples per figure; mean value (pixel) and coefficient of variation (CV)].

**No**.	**Calculation**		**Assumed derived information to herd manager**	
			**Camera-based assessment (p.m.)**	**Manual assessment on-farm**
1	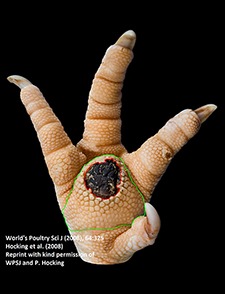	Green line: 226953.3 Pixel (CV 0.002) red line: 57076.7 Pixel (CV 0.004) = 25.1%	Camera-based result - Score “2” or “3” - Considering Hocking: Score 2 necrosis up to 25% of foot pad, Score 3 necrosis between 25 and 50% of foot pad. - Original scheme classified Score “4” (more than 50% necrotic area).	- Major problem in flock. - Measures are necessary immediately.
2	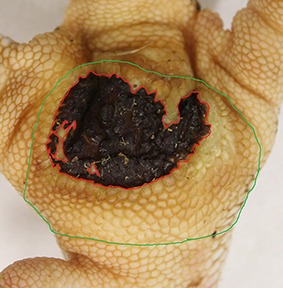	Green line: 3556313 Pixel (CV 0.002) red line: 1322907 Pixel (CV 0.001) = 37.2 %	Camera-based result - Score “3.”	- Major problem in flock. - Measures are necessary immediately.
3	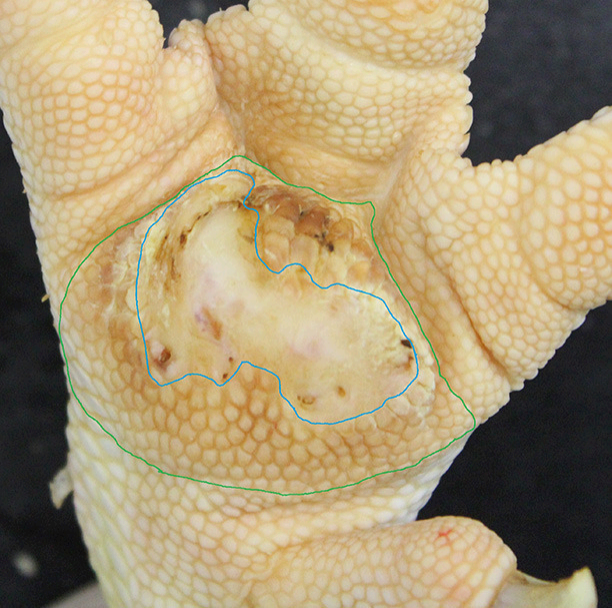	Green line: 2038920 Pixel (CV 0.000) blue line: 1063746 Pixel (CV 0.002) = 52.2 %	Camera-based result - Score “0.” Even though white scar tissue is present, that only provides information of an older, partly healed process. Lost information p.m: - Problems during husbandry period. - Successful measure to improve foot pad health.	- There was a problem in stock, bedding material/ litter moisture. - Successful measure improved foot pad health.
4	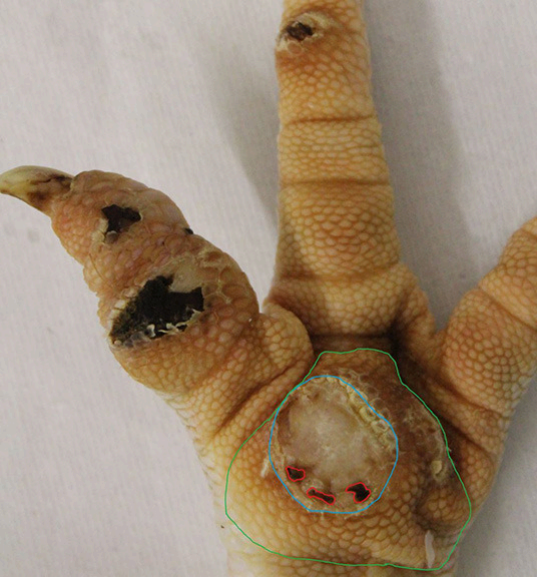	Green line: 1102192 Pixel (CV 0.004) red line: 24500 Pixel (CV 0.000) = 2.2% blue line: 416407 Pixel (CV 0.002) = 35.6%	Camera-based result - Score “1.” Even though white scar tissue is present, that only provides information of an older, partly healed process Lost information p.m. - Problems during husbandry period. - Successful measure to improve foot pad health.	- Severe lesions on digital pads. - Lessen necrosis. - Scar tissue on metatarsal pad.

The foot in the second photographic image was subjectively classified as being affected by a huge necrotic area. The red area was calculated by the image program to have an altered surface area of 37.2%. That would correspond to a camera-based Score 3 when following the Hocking System (Score 0—no lesions to Score 4—necrotic lesions > 50% of foot pad) after slaughtering (1). Again, three-dimensionality was not considered. The third and fourth photographic images show the formation of scar tissue over an area calculated to be 52.2 and 35.6% of the foot pad, respectively.

### Discussion

Several foot pad alterations were quantified in proportion to the perceived foot pad area of the metatarsal pad. This highlights the lack of an anatomic and macroscopic definition of the metatarsal pad. The size is not clear and obviously too large in relation to the necrotic area. Furthermore, the method used does not consider three-dimensionality, which would probably lead to an increase in the proportion of the non-affected area. The optical illusion becomes clear in the second photographic image (No. 2). Based on manual assessment, the necrotic area would probably be within the range equal to Score 4, while the result from camera-based assessment would diminish FPD severity to a computer-based Score 3. On-farm foot pad assessment enables the immediate implementation of management measures. Therefore, further discussion is probably required concerning the percentage of the necrotic area which categorizes a foot pad with severe lesions (<50%) which, in accordance with health programs, could have consequences for obviously high-risk turkey farms. Furthermore, Mayne et al. (3) and Youssef et al. (17) discovered visible cellular changes on foot pads after continuous exposure to wet litter over a period of 48 or 8 h per day. The process of healing took about 15 days when young turkeys were transferred from wet (26% dry matter content) to dry litter (87% dry matter content) (3). Suitable litter material, other than straw, could be sawdust or wood shavings which seem to result in less caked litter. However, a coarse litter material structure must also be considered (3, 14, 35). Rudolf (33) observed the correlation between litter material and the formation of scar tissue. Toms and hens which were kept on wood shavings showed a higher formation of scar tissue on the metatarsal pads, compared to birds which were kept on unchopped straw. Additionally, Platt (18) found that the highest prevalence of scar tissue was observed between the 14th and 21st week of life, during on-farm assessment. When taking these results into consideration, they suggest the necessity of monitoring first alterations as well as scar formation on foot pads. Due to the importance of management measures with a focus on dry litter material, quick and prompt action is needed in order to combat prevention of severe foot pad lesions and receive feedback on successful management. Camera-based analysis focusing on the size of the necrotic area currently takes neither alteration on digits, nor scar tissue into account. This information would be important to inform any need for management measures in subsequent flocks. This would not only increase the importance of on-farm foot pad assessment but also require a scoring system which categorizes scar formation and digits separately from lesions of the metatarsal pad.

### Overall Conclusions

In conclusion, an on-farm foot pad scoring system is necessary to improve foot pad health in turkey husbandry. A 4-week evaluation interval would match the time for formation of scar tissue and allow for reflection on the success and necessity of management measures. To increase welfare levels on farms, both bird feet should be monitored, and the most affected foot should be evaluated. The scoring system used should consider first alterations separately from non-affected feet, as well as digital pads from metatarsal pads and also the formation of scar tissue. Further studies are also required to fill the gap on the boundary of the metatarsal pad and the histopathological findings. Finally, the applied scoring system would require a more detailed scale, especially up to 50% macroscopic alterations on the plantar foot pad. An important issue, necessary for the improvement of FPD assessment validity, is an external standard that supports the comparability of foot pad results. Implementing this statement on the recovery rate would enable a comparison to be made between foot pad findings from manual and automatic assessments (36).

## Data Availability

The raw data supporting the conclusions of this manuscript will be made available by the authors, without undue reservation, to any qualified researcher.

## Ethics Statement

The study was carried out in accordance with the German legislations, the German Animal Protection Act (37), the “National reference figures for a voluntary agreement on keeping fattening turkeys” (38) and national requirements for animal husbandry (39) as well as the “Guide for the Care and Use of Agricultural Animals in research and Teaching” (40). Data from Section 1 were collected within a project from the “Animal Welfare Plan of Lower Saxony” (36). The studies were supported and monitored by officials from the Lower Saxony Ministry of Food, Agriculture and Consumer Protection and the Lower Saxony State Office for Consumer Protection and Food Safety (LAVES). Turkey feet for Section 3 were collected after a commercial slaughtering process at a German slaughterhouse.

## Author Contributions

KT, BS, and RA conceived the study. KT and BS processed the experimental data, performed the analysis and designed the figures in Sections 1 and 3. BS and NK prepared and executed parts of the histopathological section. MG and RA provided support and advice. KT wrote the manuscript in consultation with BS, FK, MG, NK, and RA. All the authors contributed to the revision of the manuscript and approved the final version of the manuscript for submission.

### Conflict of Interest Statement

The authors declare that the research was conducted in the absence of any commercial or financial relationships that could be construed as a potential conflict of interest.
